# Supplementary material for: The most active Cu facet for low-temperature water gas shift reaction
Source: Nat Commun. 2017 Sep 8;8:488. doi: 10.1038/s41467-017-00620-6 (PMC5591213; doi:10.1038/s41467-017-00620-6)
Supplement: Supplementary file 1 — Supplementary Information [file 41467_2017_620_MOESM1_ESM.pdf]

Description of Supplementary Files

File name: Supplementary Information
Description: Supplementary figures, supplementary tables, supplementary notes, supplementary methods and supplementary references.

File name: Peer review file

Supplementary Methods

Elemental compositions were analyzed with an Optima 7300 DV inductively coupled plasma atomic emission spectrometer (ICP-AES).Powder X-ray diffraction (XRD) patterns were recorded on a Philips X'Pert PROS diffractometer using a nickel-filtered Cu Kα (wavelength: 0.15418 nm) radiation source with the operation voltage and operation current being 40 kV and 50 mA, respectively. Routine X-ray photoelectron spectroscopy (XPS) measurements were performed on an ESCALAB 250 high performance electron spectrometer using monochromatized Al Kα (hν = 1486.7 eV) as the excitation source. The likely charging of samples was corrected by setting the binding energy of the adventitious carbon (C1s) to 284.8 eV. Scanning electron microscope (SEM) experiments were performed on a JEOL JSM-6700 field emission scanning electron microscope. Transmission electron microscopy (TEM), high resolution transmission electron microscopy (HRTEM) and selected area electron diffraction (SAED) experiments were performed on a JEM-2100F high resolution transmission electron microscope. BET specific surface areas were acquired by using a Beckman Coulter SA3100 surface area analyzer, and the sample was degassed at 423 K for 5 h in a N_2 atmosphere before the measurements.

Experiments of CO temperature-programmed reduction (CO-TPR), temperature-programmed reaction spectra (TPRS) and temperature desorption spectra

(TDS) were conducted on a Micromeritics ChemiSorb 2750 connected with an online HIDEN QIC-20 gas analysis system to detect the compositions of the effluent gas. In CO-TPR experiments, 0.01 g Cu_2O NCs were placed in a U-shaped tube and headed at the rate of 5 K·min^{-1} in a 5% CO/Ar atmosphere with a flow rate of 40 mL·min^{-1}. In CO+H_2O-TPRS experiments, 0.1 g Cu NCs were acquired by the in-situ reduction of Cu_2O NCs in a U-shaped tube and then heated to 548 K at the rate of 5 K·min^{-1} in a gas stream consisting of 0.432% CO and water vapor at 278 K (water vapor: 0.8726 kPa) balanced with Ar at a flow rate of 30 mL·min^{-1} to yield the P_{CO}:P_{H2O} ratio of 1:2 and kept for 0.5 h. In TDS experiments, 0.1 g Cu NCs subjected to the catalytic activity evaluation in the WGS reaction up to 548 K were placed a in U-shaped tube and heated in a high-pure Ar stream at a flow rate of 30 mL·min^{-1} to 873 K at a rate of 5 K·min^{-1}.

XPS experiments without exposure to air were carried out on an ESCALAB 250 high performance electron spectrometer equipped with an high-pressure reactor using monochromatized Al Kα (hν = 1486.7 eV) as the excitation source. The samples were treated under desired conditions at ambient pressure in the high-pressure reactor and then cooled to room temperature, pumped and transferred to the analysis chamber for the XPS measurements without exposures to air. The Cu_2O NCs were heated to 548 K at a rate of 1 K·min^{-1} and kept for 2 h in 5% CO/Ar (flow rate: 30 mL·min^{-1}) to acquire Cu NCs. The condition of CO+H_2O reaction on Cu NCs employed in the XPS experiments was same as

that employed in the $CO+H_2O$-TPRS experiment. During the H_2O activation experiments, Cu NCs were heated to the desirable temperatures at a rate of 5 $K \cdot min^{-1}$ in the Ar stream (flow rate: 30$mL \cdot min^{-1}$) saturated with water vapor at 278 K and kept for 0.5 h. The Cu NCs subjected to water activation at 523 K were heated to 473 K at a rate of 5 $K \cdot min^{-1}$ in a 0.432% CO/Ar stream at a flow of 30 $mL \cdot min^{-1}$ and kept for 0.5 h to the reactivity of water-activated Cu NCs toward CO.

In-situ Diffuse reflectance infrared Fourier transformed spectroscopy (DRIFTS) experiments were carried out on a Nicolet 6700 FT-IR spectrometer equipped with an in-situ low-temperature and high-vacuum DRIFTS reaction cell (Harrick Scientific Products, INC) using an MCT/A detector in the series mode with 256 scans and at a resolution of 4 cm^{-1}. 0.1 g Cu_2O NCs were loaded on the sample stage of the reaction cell and heated in 5% CO/Ar (flow rate: 30 $mL \cdot min^{-1}$) to 548 K at a rate of 1 $K \cdot min^{-1}$ and kept for 2 h to acquire Cu NCs. During CO adsorption experiments, the Cu NCs were purged in Ar at room temperature for 0.5 h, pumped to a pressure of 0.01 Pa and then cooled to 123 K, and the spectra were taken as the background spectra. Then CO was admitted via a leak valve into the reaction cell to reach a pressure of 400 Pa, and the DRIFTS spectra were recorded after CO adsorption reached the steady state. During TDS experiments of used Cu NCs catalysts, 0.1 g Cu NCs subjected to catalytic activity evaluation in the WGS reaction were loaded on the sample stage of the reaction cell and purged in Ar at 303 K,

and the spectra were taken as the background spectra. Then the Cu NCs were heated to 723 K in Ar (flow rate: 30 $mL \cdot min^{-1}$) at a heating rate of 5 $K \cdot min^{-1}$ and kept for 1 h, and then cooled down to 303 K at which temperature the DRIFTS spectra were recorded.

Catalytic activity in the water-gas-shift (WGS) reaction was evaluated in a fixed-bed flow reactor. The reactant gas consisted of 5% CO (flow rate: 30 $mL \cdot min^{-1}$) and water vapor at 319 K (water vapor pressure: 10.094 kPa) balanced with Ar that yielded the P_{CO}:P_{H2O} ratio of 1:2. 0.1 g Cu_2O NCs diluted with 0.05 g Al_2O_3 were firstly in-situ reduced by 5% CO/Ar (flow rate: 30$mL \cdot min^{-1}$) at 548 K for 2 h to acquire Cu NCs. The acquired Cu NCs catalysts were heated to the desired reaction temperatures at a rate of 1 $K \cdot min^{-1}$ and the steady-state compositions of the effluent gas were analyzed with an on-line gas chromatography (FULI 9790II) with a thermal conductivity detector attached to a 5A zeolite column. The catalytic activity was calculated by the change in CO concentration of the inlet and outlet gases. The catalytic activity of Cu_2O NCs was evaluated using the same procedure as Cu NCs but without the pretreatment of reduction. The catalytic activity of ZnO/Cu NCs catalysts were evaluated using the same procedure as Cu NCs employing 0.05 g ZnO/Cu_2O NCs composites diluted with 0.1 g Al_2O_3 pre-reduced by 5% CO/Ar (flow rate: 30 $mL \cdot min^{-1}$) at 423 K for 1 h to acquire ZnO/Cu NCs catalysts. The catalytic activity of commercial $Cu/ZnO/Al_2O_3$ catalyst was evaluated

using the same procedure as Cu NCs employing 0.005 g Cu/ZnO/Al_2O_3 catalyst diluted with 0.145 g Al_2O_3 carefully pre-activated in H_2.

DFT calculations were carried out by the Vienna *ab-initio* simulation package (VASP)[5,6] using the projector-augment wave (PAW) method.[7] The nonlocal exchange correlation energy was performed via the generalized gradient approximation (GGA) and PBE functional.[8,9] A plane wave basis set with a 400 eV cut off kinetic energy and a 5×5×1 Monkhorst-Pack k-point grid to sample the surface Brillouin zone were performed to give converged results. Slab model was used to simulate the Cu_2O/Cu(111) andCu_2O/Cu(100) structures with a vacuum layer of 15 Å, built from the fcc-Cu bulk unit cell with an optimized lattice parameter of 3.638Å that agrees well with the experimental value of 3.615 Å.[10] Cu(111) and Cu(100) surfaces respectively had the thickness of 3 and 4 atom layers with a 2×4 periodic supercell. The Cu_2O structure adopted the copper configurations of Cu(111)/(100) substrates with a 2×2 periodic supercell and consisted of an O_{down}-Cu-O_{up} layer. According to previous results,[11-14] ring-like structures were adopted for Cu_2O islands on both Cu(100) and Cu(111) surfaces, which gives a zig-zag chain structure of Cu_2O at the Cu-Cu_2O interface. Thus, to simulate the reaction at the interface between Cu and Cu_2O domain but remain computational affordable to explore full reaction network, we use a zig-zag chain structure representing the edge of Cu_2O ring-like structures. Moreover, the lattice constant of Cu_2O ring-like structure was assumed same with Cu metal underneath

for simplicity. The top layer of Cu(111) , top two layers of Cu(100) substrates, the whole Cu_2O-like layer, and the adsorbed species were allowed to relax until the force on each ion was less than 0.02 eV Å^{-1} during the optimization processes.

The adsorption energies of adsorbates (E_{ads}) were calculated as

$$E_{ads} = E_{ad/sub} - E_{ad} - E_{sub}$$

in which $E_{ad/sub}$ is the energy of the optimized adsorption system of adsorbate and substrate, E_{ad} is the energy of adsobate in the gas phase and E_{sub} is the energy of the clean substrate.

The reaction energies of elementary reactions (E_r) were calculated as

$$E_r = \sum(E_{ads})_{products} - \sum(E_{ads})_{reactants} + \Delta E_{gas\ phase}$$

where $\sum(E_{ads})_{products}$ and $\sum(E_{ads})_{reactants}$ are the total energies of the adsorption energies of the products and reactants at infinite separation. $\Delta E_{gas\ phase}$ is the corresponding reaction energy in the gas phase. Therefore, negative values of E_r mean exothermic processes, and positive values of E_r mean endothermic processes.

To search the transition states of elementary reactions, we combined the climbing-image nudged elastic band (CI-NEB) method[15,16] with the advanced force reversed method[17] and the convergence criteria for the transition state calculation was that the force on each atom decreased to 0.05eV Å^{-1}. The transition states were verified by vibrational analysis showing a single imaginary mode. Zero point energies and entropy corrections were neglected in DFT calculations. The activation energies of elementary

reactions (Ea) and reaction energies of elementary reactions (E_r) were taken with respect to isolated reactants/products. Four typical elementary surface reactions were selected to calculate the interaction energy between isolated species and co-adsorbed states. The results are listed as the following:

Elementary Surface Reactions	States	Interaction Energy (eV)	
		(111)	(100)
$H_2O_{Cu} + O_{Cu2O} \rightarrow OH_{Cu} + O_{Cu2O}H$	Final State<br>$OH_{Cu} + O_{Cu2O}H$	0.47	0.41
$HCOO_{Cu2O} \rightarrow CO_{2, Cu2O} + H_{Cu}$	Final State<br>$CO_{2, Cu2O} + H_{Cu}$	0.05	0.08
$H_{Cu} + H_{Cu} \rightarrow H_2(g)$	Initial State<br>$H_{Cu} + H_{Cu}$	0	0
$CO_{Cu} + O_{Cu2O}H \rightarrow COOH_{Cu2O}$	Initial State<br>$CO_{Cu} + O_{Cu2O}H$	0.07	-0.02

It can be found that most of them have modest interaction energies (less than 0.09 eV), and therefore can be neglected. The small interaction might come from the relative large supercell (2×4) used in present work. Large interaction energies were observed for water dissociation at Cu_2O-Cu interfaces, being about 0.47 eV for Cu_2O-Cu(111) and 0.41 eV for Cu_2O-Cu(100). This comes from the cost of hydrogen bond between dissociated fragments at the co-adsorbed states.

Supplementary Notes

The absence of mass and heat transfer limitations under our catalytic reaction conditions was proved by a Weisz-Prater analysis and a Mears analysis proposed by Oyama *et al.*[1] as the following:

Supplementary Note 1. Mass transfer limitations: Internal diffusion, Weisz-Prater Criterion[2,3]

The absence of internal mass transfer limitations was evaluated using the Weisz-Prater criterion, where if C_{WP} is lower than 1, the internal mass transfer effects can be neglected:

$$C_{WP} = \frac{-r'_{A,Obs}\,\rho_c R^2}{D_e C_{AS}} < 1$$

$-r'_{A,Obs}$, Observed reaction rate = 3.15×10^{-6} kmol kg_{cat}^{-1} s^{-1} (taking the maximum CO rate observed in our investigations)

ρ_c, Solid density of catalyst = 6000 kg m^{-3}

R, Particle size = 3.75×10^{-5} m

C_{AS}, Concentration of reactant A on the surface. As the maximum concentration (5 vol%) used considering all kinetic experiments is that of A = Carbon monoxide. Considering 5 vol% CO, C_{AS}= 2.04×10^{-3} kmol m^{-3}

D_e, effective diffusion given by $D_e = \frac{D_{AB}\varepsilon_p\sigma_c}{\tau}$

D_{AB}, Gas-phase diffusivity. For a mixture of CO-Ar was calculated[3] to be 5.61×10^{-6} m^2 s^{-1}, ε_p, Pellet porosity = 0.4; σ_c, Constriction factor = 0.8; τ, Tortuosity = 3; D_e = 5.98×10^{-6} m^2 s^{-1}

C_{WP} = [(3.15×10^{-6})×6000×(3.75×10^{-5})^2]/[(5.98×10^{-6})×(2.04×10^{-3})] = 2.17×10^{-2} < 1

Therefore, this system does not suffer from internal mass transfer limitations.

Supplementary Note 2. Mass transfer limitations: External Diffusion, Mears Criterion[2]

The absence of external mass transfer limitations can be evaluated using the Mears criterion:

$$C_M = \frac{-r'_{A,}\rho_b R n}{k_c C_{Ab}} < 0.15$$

$-r'_{A,Obs}$, Observed reaction rate = 3.15×10^{-6} kmol kg$_{cat}^{-1}$ s^{-1} (taking the maximum CO rate observed in our investigations)

ρ_c, Solid density of catalyst = 398.089 kg m^{-3}

R, Particle size = 3.75×10^{-5} m

n, reaction order = 1

C_{Ab}, Bulk of CO. If CO is 5 %, C_{AS}= 2.04×10^{-3} kmol m^{-3}

k_c mass transfer coefficient = 0.0174 m s^{-1}

C_M = [(3.15×10^{-6}) × 398.089 × (3.75×10^{-5}) × 1]/[(0.0174) × (2.04×10^{-3})] = 1.32 x10^{-4} <<1

Therefore, this system does not suffer from external mass transfer limitations.

Supplementary Note 3. External (Interphase) heat transfer: Mears criterion[2]

$$\left|\frac{-\Delta H_r(-r_A')\rho_b RE}{h_t T_b^{\,2} R_g}\right| < 0.15$$

$-\Delta H_\mathrm{r}$, heat of reaction, -41.2 kJ mol^{-1} (taking the maximum heat of WGS reaction)

$-r_\mathrm{A}'$, reaction rate, 3.15×10^{-3} mol kg$_{cat}$$^{-1}$ s^{-1} (taking the maximum CO rate observed in our investigations)

ρ_b, bulk density of catalyst bed, 398.089 kg m^{-3}

R, catalyst particle radius, 3.75×10^{-5} m

E, activation energy, 32.4 kJ mol^{-1}

h_t, heat transfer coefficient between gas and pellet, 0.747 kJ m^{-2}·K^{-1} s^{-4}

T_b, bulk gas temperature, 548 K

R_g, gas constant, 8.3145×10^{-3} kJ mol^{-1} K^{-1}

[41.2 kJ mol^{-1}] × [3.15×10^{-3} mol kg$_{cat}$$^{-1}$ s^{-1}] × [398.089 kg m^{-3}] × [3.75×10^{-5} m] × [32.4 kJ mol^{-1}] / ([0.747 kJ m^{-2} K^{-1} s^{-1}] × [548 K]^2 × [8.3145 ×10^{-3} kJ mol^{-1} K^{-1}]) = 3.37 x 10^{-5} << 0.15

Supplementary Figures

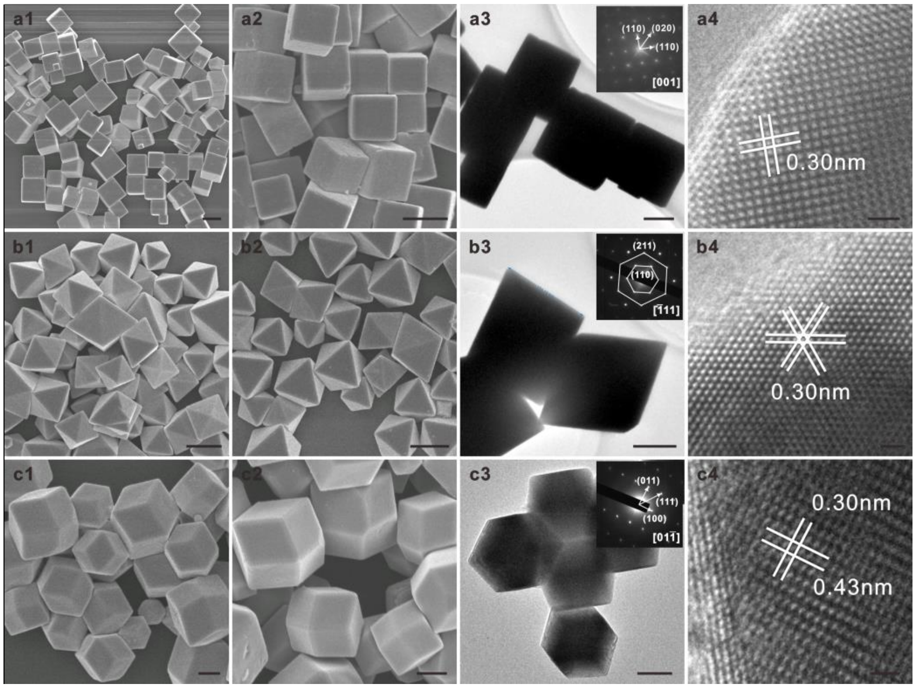

Supplementary Figure 1. The scale bars of (**a1, a2, b1 and b2)** correspond to 1000 nm, that of (**a3, b3 and c1-c3**) correspond to 500 nm, and that of (**a4-c4**) correspond to 1 nm. (**a1-a4**) Representative SEM, TEM, and HRTEM images of c-Cu_2O NCs, (**b1**) representative SEM image of as-synthesized PVP capped o-Cu_2O-PVP NCs, (**b2-b4**) representative SEM, TEM, and HRTEM images of capping-ligand-free o-Cu_2O NC,(**c1**) representative SEM image of as-synthesized OA capped d-Cu_2O-OA NCs, and (**c2-c4**) representative SEM, TEM, and HRTEM images of capping-ligand-free d-Cu_2O NCs. Lattice fringes of 0.30 and 0.43 nm respectively correspond to the spacing of Cu_2O {110} and {200} crystal planes (JCPDS card NO. 78-2076). The insets in Supplementary Figure 1 a3-c3 show the electron diffraction patterns. These results demonstrate that the

morphologies of as-synthesized o-Cu_2O-PVP and d-Cu_2O-OA NCs do not changed during the controlled oxidation treatment to acquire capping-ligand-free o-Cu_2O and d-Cu_2O NCs, respectively.

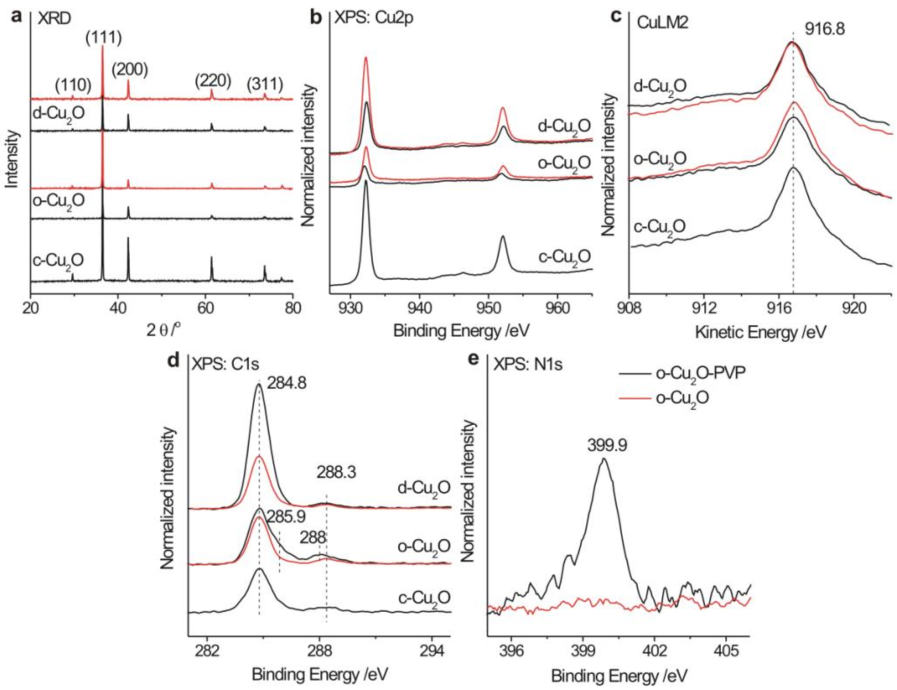

Supplementary Figure 2. (**a**) XRD, (**b**) Cu 2p XPS, (**c**) Cu LMM AES, (**d**) C 1s and (**e**) N 1s XPS spectra of as-synthesized Cu_2O NCs (black lines) and capping-ligand-free Cu_2O NCs (red lines). In the C 1s XPS spectra, the C 1s peaks at 285.9 and 288 eV respectively corresponding to the C=O group and the C-N group of the PVP molecules on o-Cu_2O-PVP disappeared after the controlled oxidation treatment, and the C 1s peak at 284.8 eV corresponding to the adventitious carbon species/C atoms in CH_x groups of the PVP molecules attenuated; and meanwhile, in the N 1s XPS spectra, the N 1s peak at 399.9 eV corresponding to the C-N group of the PVP molecule disappeared. In the C 1s XPS spectra, the C 1s peak at 284.8 eV belonging to the adventitious carbons species/C atoms in the CH_x groups of the OA molecule on d-Cu_2O-OA greatly attenuated. XRD patterns and Cu 2p XPS and LMM AES spectra exhibited the same features for as-synthesized Cu_2O NCs and

capping-ligand-free Cu_2O NCs. These results demonstrate that the employing controlled oxidation treatments successfully remove the PVP and OA capping ligands respectively on as-synthesized o-Cu_2O-PVP and d-Cu_2O-OA while both the bulk and surface structures of Cu_2O NCs do not change, agreeing with our previous reports.

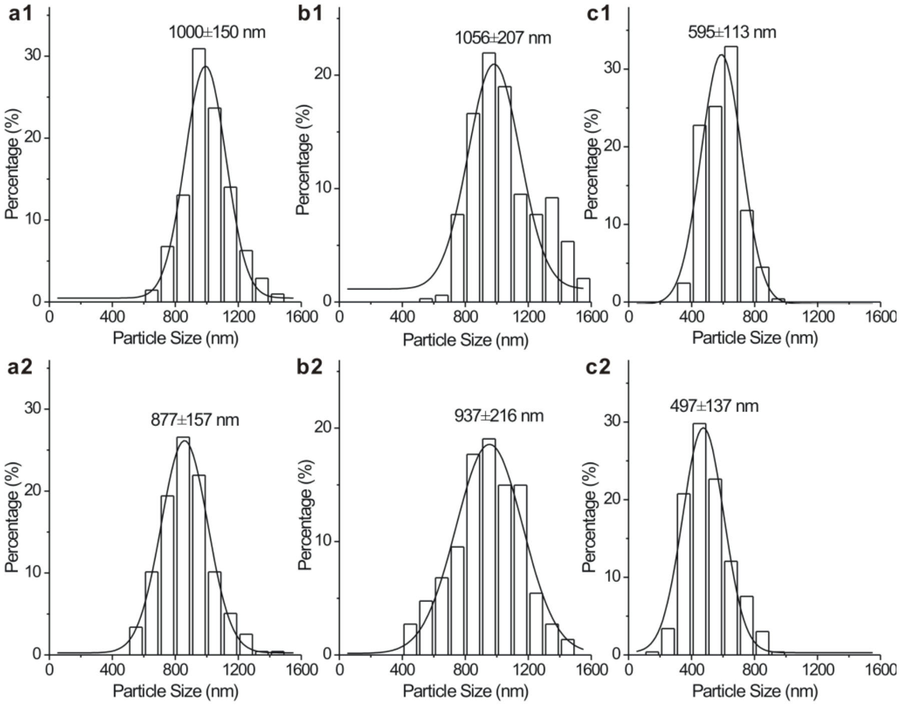

Supplementary Figure 3. Size distributions of (**a1**) c-Cu_2O, (**a2**) c-Cu, (**b1**) o-Cu_2O, (**b2**) o-Cu, (**c1**) d-Cu_2O and (**c2**) d-Cu.

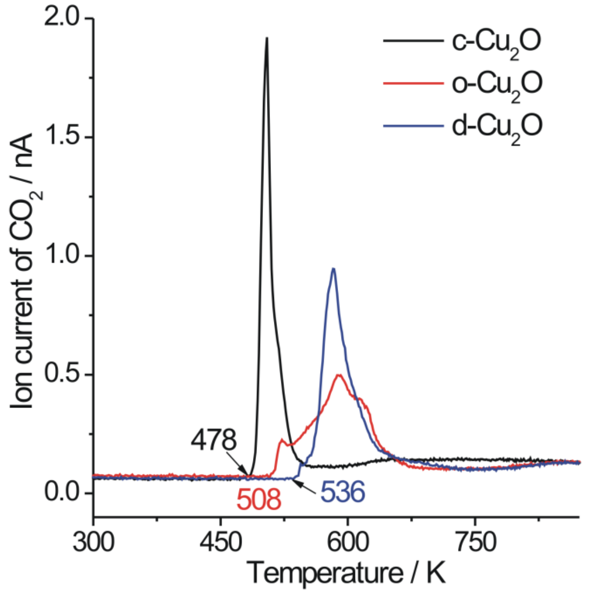

Supplementary Figure 4. CO-TPR profiles of c-Cu_2O, o-Cu_2O, and d-Cu_2O NCs. It can be seen that all Cu_2O NCs can be reduced at 548 K.

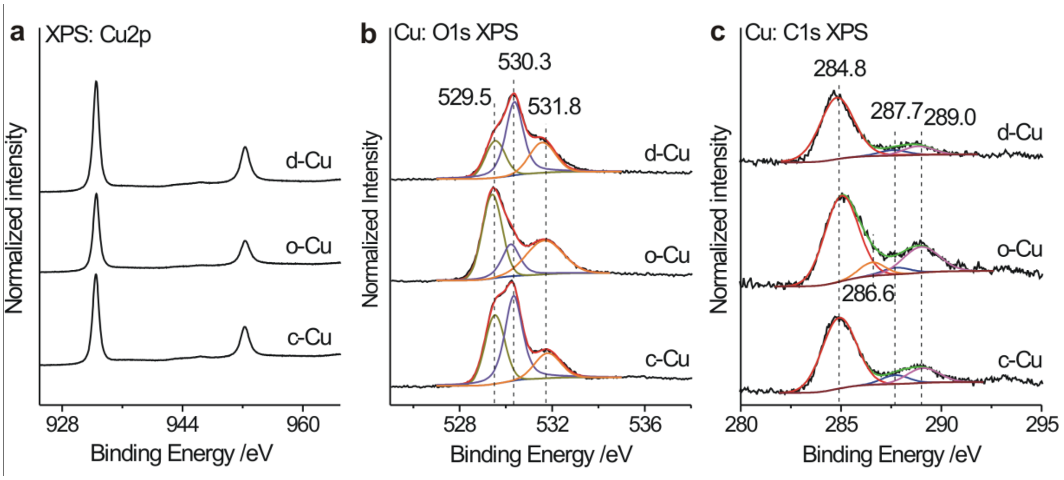

Supplementary Figure 5. XPS spectra measured without exposure to air with peak-fitting results of (**a**) Cu 2p, (**b**) O 1s and (**c**) C 1s of various Cu NCs. The thin solid line and thick solid lines correspond respectively to original data and peak-fitted spectra. In the Cu 2p XPS spectra no Cu(II) feature was observed. In the O 1s XPS spectra, three components appear with the O 1s binding energy at 529.5, 530.3, and 531.8 eV that could be respectively assigned to oxygen adatoms, Cu suboxide, and hydroxyl groups/oxygenates species; in the C 1s XPS spectra, adventitious carbon, carbonate and carboxylate species with the C 1s binding energy respectively at 284.8, 289 and 287.7 eV are visible for all Cu NCs while minor formate species with the C 1s binding energy at 286.6 eV exists exclusively for o-Cu.

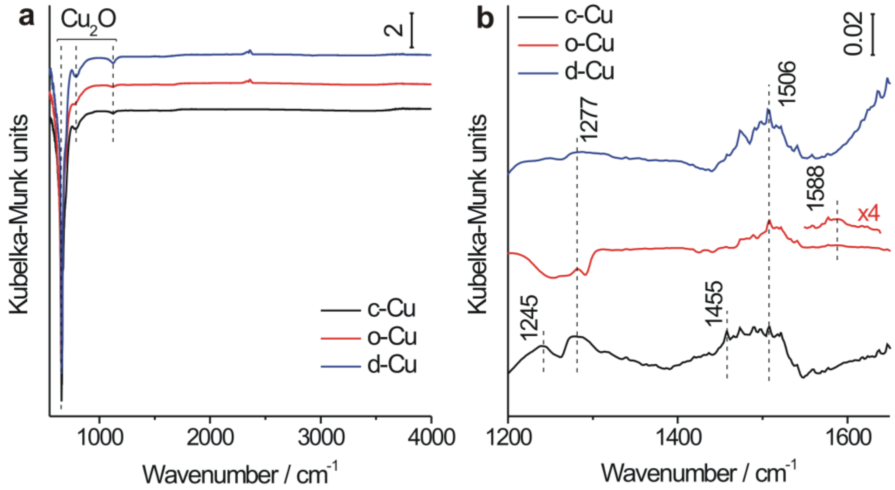

Supplementary Figure 6. (**a**) In-situ DRIFTS spectra of Cu NCs acquired by the reduction of corresponding Cu_2O NCs with the spectrum of corresponding Cu_2O NCs as the reference spectrum and (**b**) the enlarge image between 1200 and 1650 cm^{-1}.

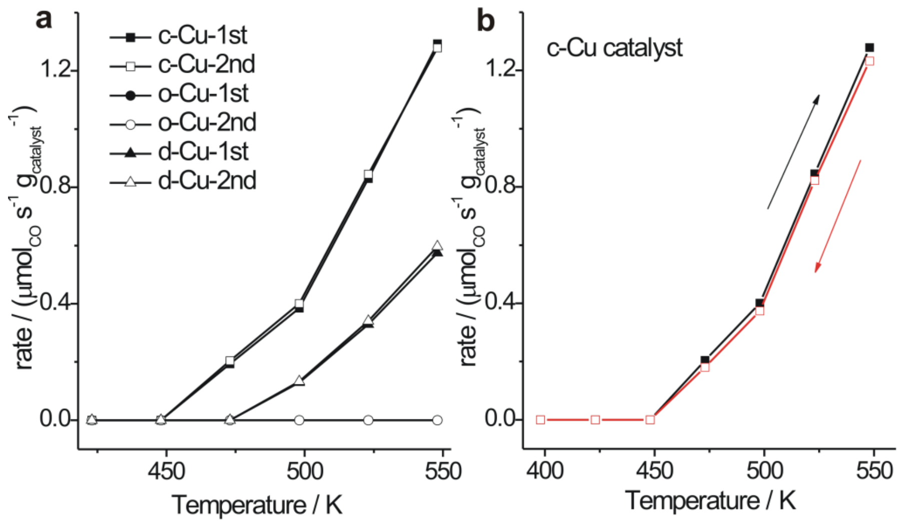

Supplementary Figure 7. (**a**) Catalytic performance of Cu NCs in the WGS reaction evaluated for two cycles and (**b**) Catalytic performance of c-Cu NCs in the WGS reaction evaluated with cycling reaction temperatures. The observed reproducibility demonstrated the stability of Cu NCs in the WGS reaction up to 548 K. With the specific surface areas of c-Cu (1.17 $m^2 \cdot g^{-1}$) and d-Cu NCs (3.33 $m^2 \cdot g^{-1}$) and the Cu atom densities of Cu (100) (1.51167×10^{19} Cu/m^2) and (110) (1.06873×10^{19} Cu/m^2) surfaces, the surface Cu atom-specific reaction rates (mol_{CO} s^{-1} $mol_{surf\ Cu}^{-1}$) of Cu NCs were calculated from corresponding reaction rates.

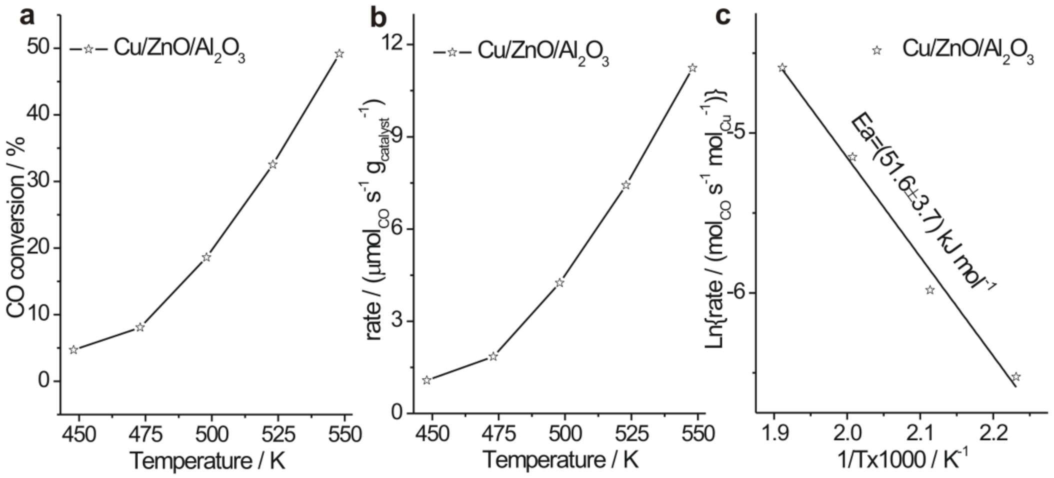

Supplementary Figure 8. (**a, b**) Catalytic performance of commercial $Cu/ZnO/Al_2O_3$ catalyst in the WGS reaction and (**c**) the corresponding Arrhenius plot.

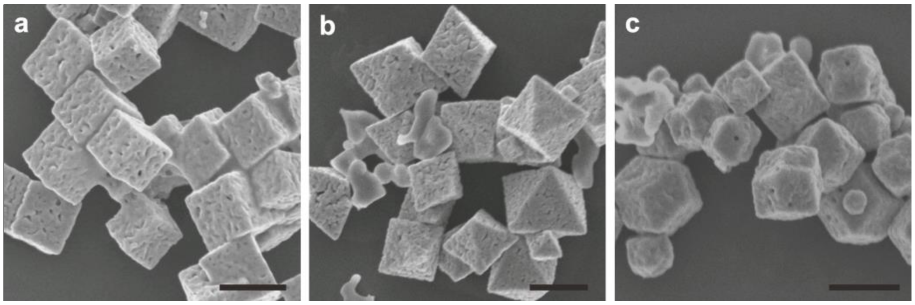

Supplementary Figure 9. The scale bars of (**a-c**) correspond to 1000 nm. SEM images of (**a**) c-Cu, (**b**) o-Cu, and (**c**) d-Cu NCs after the activity evaluation in the WGS reaction up to 548 K (Cu NCs mixed with Al_2O_3).

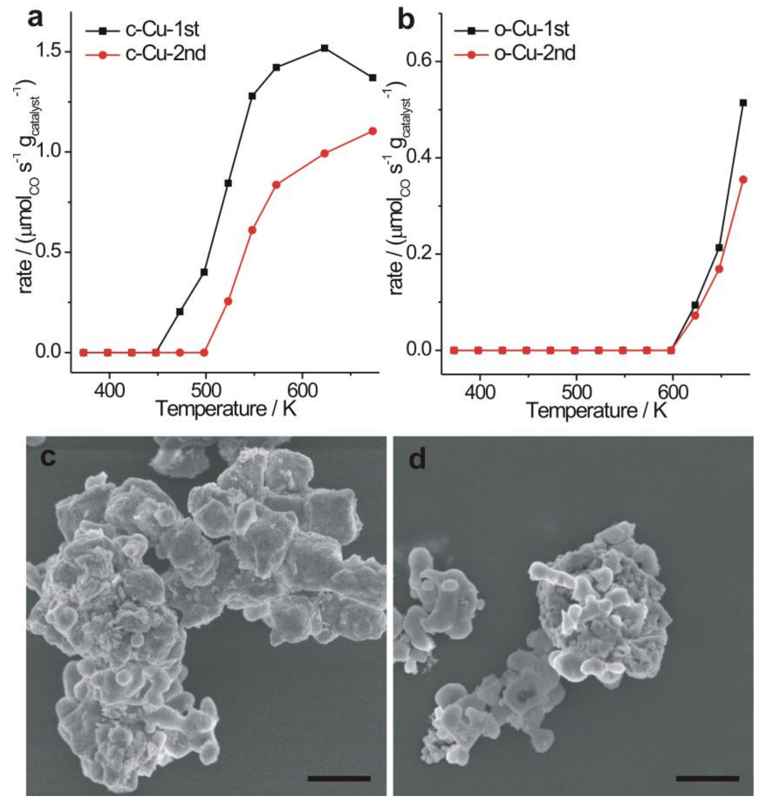

Supplementary Figure 10. The scale bars of (**c, d**) correspond to 1000 nm. Catalytic performance of (**a**) c-Cu NCs and (**b**) o-Cu NCs in the WGS reaction up to 673 K evaluated for two cycles; SEM images of (**c**) c-Cu and (**d**) o-Cu after the activity evaluation in the WGS reaction up to 673 K (Cu NCs mixed with Al_2O_3). Both Cu NCs are demonstrated not stable and undergo obvious morphological changes during the activity evaluation.

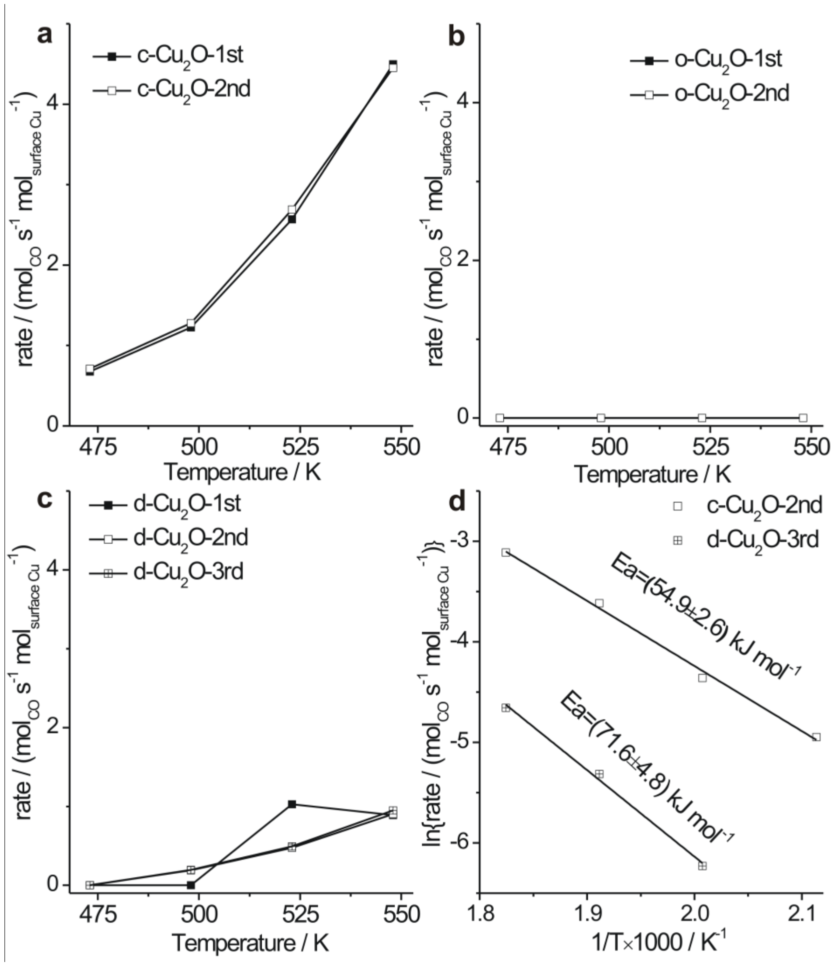

Supplementary Figure 11. Catalytic performance of (**a**) c-Cu_2O, (**b**) o-Cu_2O and (**c**) d-Cu_2O in the WGS reaction; (**d**) The Arrhenius plots of the steady-state WGS reaction catalyzed by c-Cu_2O (c-Cu_2O-2nd) and d-Cu_2O (d-Cu_2O-3rd) NCs. Each catalyst was evaluated for three cycles to confirm the steady-state activity. Both the steady-state catalytic activity and apparent activation energy of c-Cu_2O and d-Cu_2O NCs are almost identical to those of c-Cu and d-Cu NCs, respectively. The observed high CO conversion of d-Cu_2O-1st at 523 K is due to the CO_2 produced by the reduction of d-Cu_2O NCs by CO (See Supplementary Figure 12).

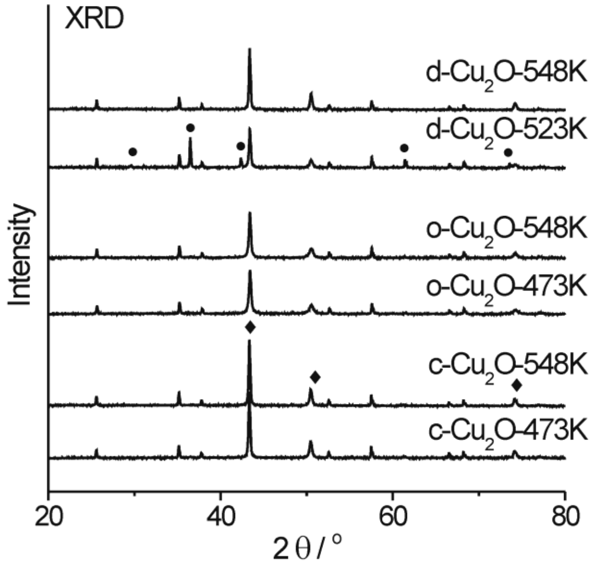

Supplementary Figure 12. XRD patterns of Cu_2O NCs catalysts during the 1st cycle of activity evaluation in the WGS reaction at selected temperature for 50 min (● Cu_2O; ◆ Cu; Others: Al_2O_3). In-situ reduction of Cu_2O NCs into Cu NCs during the WGS reaction is demonstrated. c-Cu_2O and o-Cu_2O NCs are fully reduced at temperatures not higher than 473 K while d-Cu_2O NCs are partially reduced at 523 K and fully reduced at 548 K.

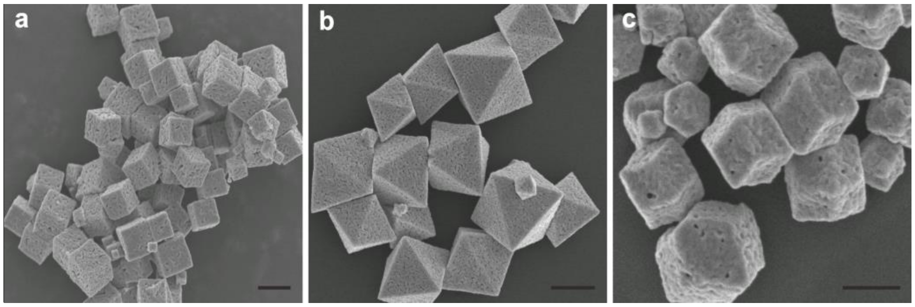

Supplementary Figure 13. The scale bars of (**a-c**) correspond to 1000 nm.

Representative SEM images of (**a**) c-Cu_2O, (**b**) o-Cu_2O and (**c**) d-Cu_2O NCs after the WGS reaction, respectively corresponding to the formation of c-Cu, o-Cu and d-Cu NCs by the in-situ reduction.

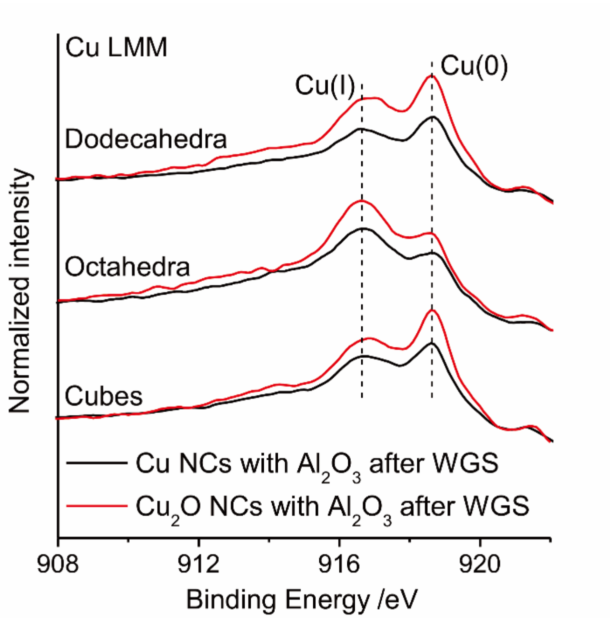

Supplementary Figure 14. Cu LMM AES spectra of Cu_2O NCs and Cu NCs mixed with Al_2O_3 after the catalytic activity evaluations in the WGS reaction up to 548 K. It can be seen that they are very similar, demonstrating that the Cu_2O NCs undergo the in-situ morphology-preserved reduction into the corresponding Cu NCs. It is noteworthy that the spectra were measured with the catalysts taken out from the reactor and exposed to air, thus the Cu(I)/Cu(0) ratios on the used Cu NCs surfaces are higher than those measured without exposure to air (Supplementary Figure 15) due to the occurrence of the surface oxidation.

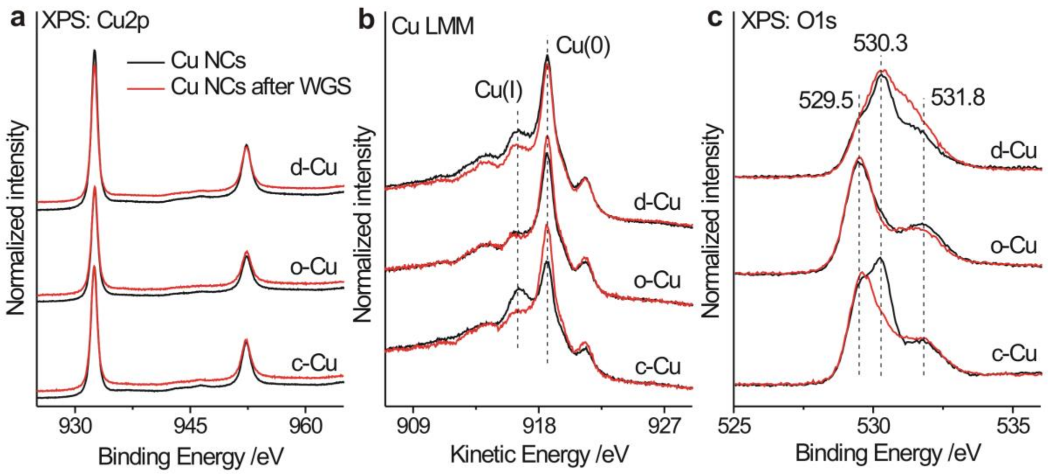

Supplementary Figure 15. (**a**) Cu 2p XPS, (**b**) Cu LMM AES, and (**c**) O 1s XPS spectra of Cu NCs before (black lines) and after (red lines) after WGS reaction at 548 K measured without exposure to air. After the WGS reaction, no Cu(II) feature appears, and the metallic Cu component increases at the expense of the Cu(I) component on used c-Cu and d-Cu due to the transformation of the suboxide species (O 1s B.E. at 530.3 eV) respectively into the oxygen adatoms (O 1s B.E. at 529.5 eV) and the hydroxyl groups/oxygenates species (O 1s B.E. at 531.8 eV) while the Cu LMM AES spectra and O 1s XPS spectra of o-Cu seldom change.

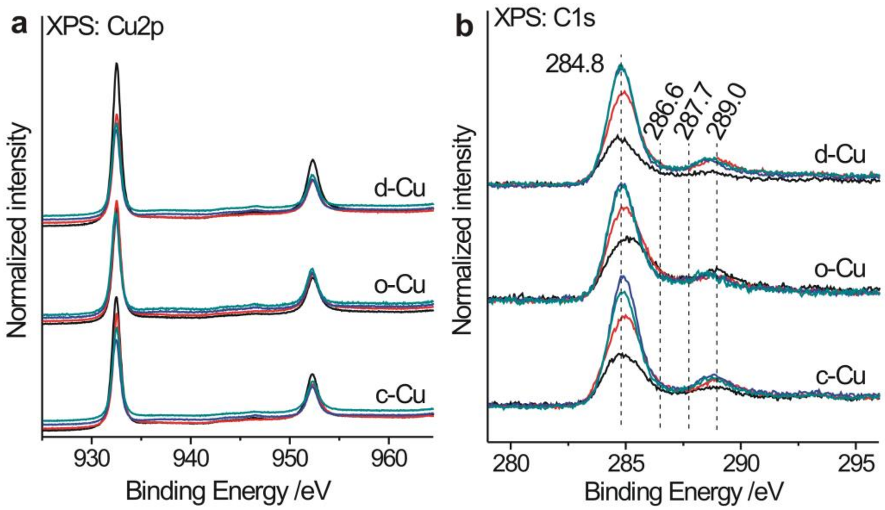

Supplementary Figure 16. (**a**) Cu 2p and (**b**) C 1s XPS spectra of Cu NCs (black lines), Cu NCs activated by H_2O at 423 K (red lines), Cu NCs activated by H_2O at 523 K (blue lines) and Cu NCs activated by H_2O at 523 K and then exposed to CO at 473 K (brown lines) measured without exposure to air.

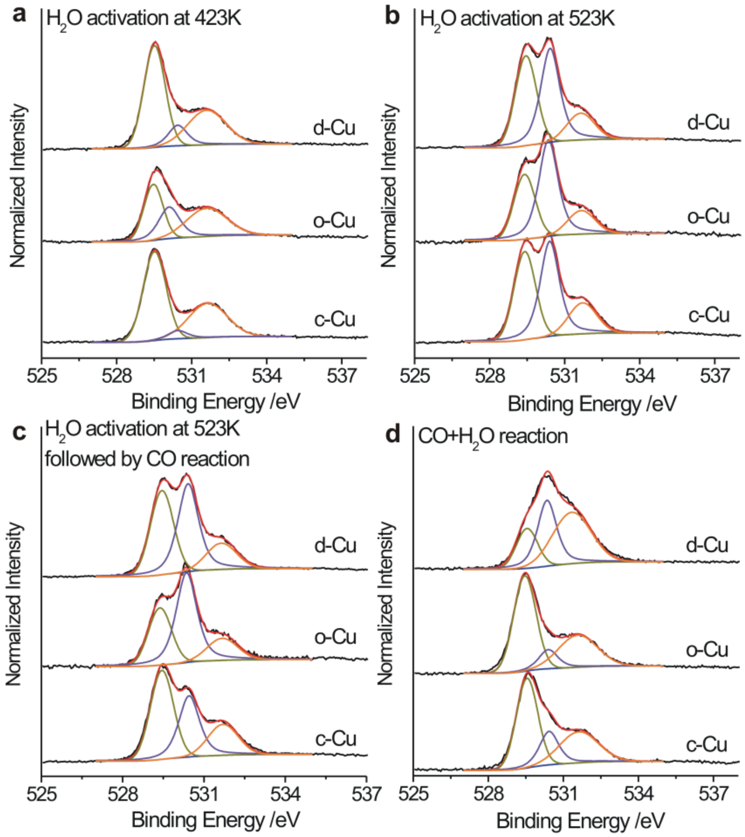

Supplementary Figure 17. O1s XPS spectra with peak-fitting results of (**a**) Cu NCs activated by H_2O at 423 K, (**b**) Cu NCs activated by H_2O at 523 K, (**c**) Cu NCs activated by H_2O at 523 K and then exposed to CO at 473 K, and (**d**) Cu NCs evaluated in the WGS reaction at 548 K measured without exposure to air.

Supplementary Table 1. The integrated peak areas of different O 1s components on Cu NCs subjected to different treatments acquired from the peak-fitting spectra of O 1s XPS spectra measured without exposure to air.

	O 1s Binding Energy (eV)								
	c-Cu			o-Cu			d-Cu		
	529.5	530.3	531.8	529.5	530.3	531.8	529.5	530.3	531.8
Cu	1.46	2.02	0.69	1.78	0.76	1.32	0.78	1.74	0.83
H_2O activation at 423 K	2.18	0.21	1.42	1.15	0.90	1.21	2.32	0.53	1.41
H_2O activation at 523 K	2.03	2.44	0.92	1.48	2.47	0.68	2.05	2.41	0.78
H_2O activation at 523 K followed by CO reaction at 473 K	2.17	1.67	0.98	1.39	2.46	0.70	2.05	2.40	0.83
WGS reaction at 548 K	1.83	0.77	1.14	2.04	0.44	1.19	0.81	1.51	1.88

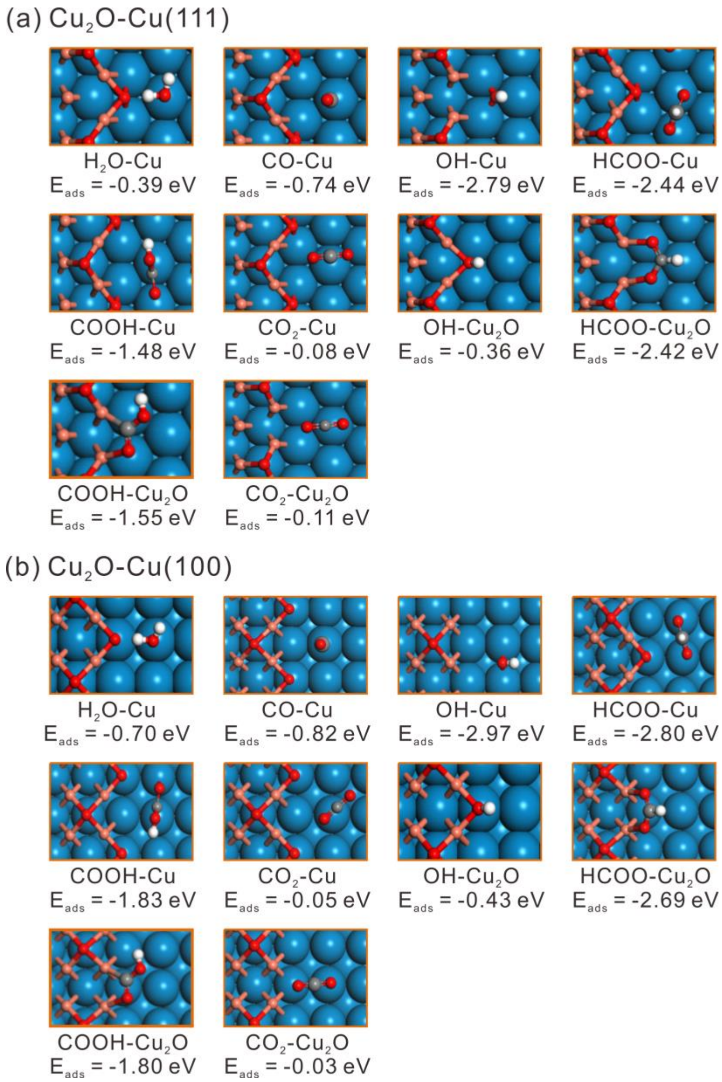

Supplementary Figure 18. The structures and the adsorption energies (E_{ads}) of adsorption species involved in WGS reaction at their favorable sites on (**a**) Cu_2O-Cu(111) and (**b**) Cu_2O-Cu(100). Labeling of atomic spheres: Cu(111)/(100) substrate in blue, Cu in Cu_2O in orange, O in red, C in grey, H in white.

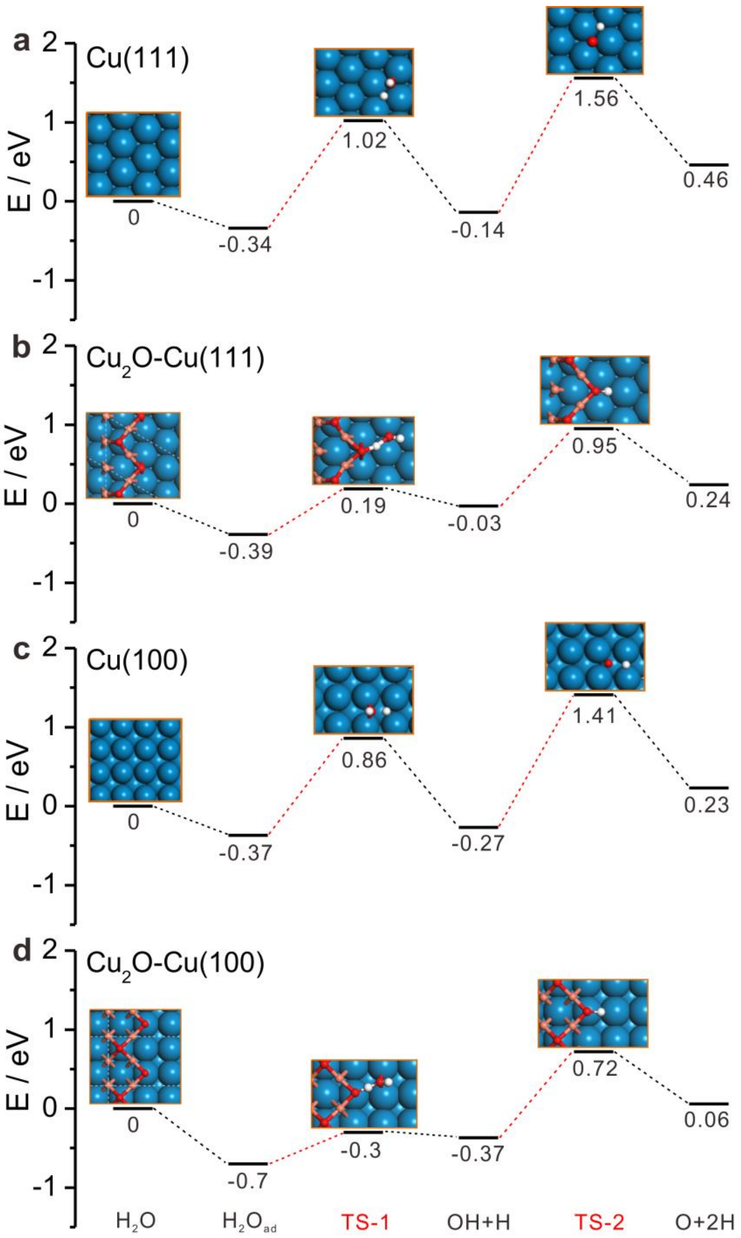

Supplementary Figure 19. Calculated energy diagram of water adsorption and decomposition on (**a**) Cu(111), (**b**) Cu_2O-Cu(111), (**c**) Cu(100) and (**d**) Cu_2O-Cu(100) surfaces with optimized structures of involved key surface species and transition states.

Supplementary Table 2. The reaction energy (E_r) and reaction barrier (E_a) of H_2O adsorption and dissociation on Cu(111), Cu(100), Cu_2O-Cu(111), and Cu_2O-Cu(100).

Elementary Reactions	Cu_2O-Cu(111)		Cu_2O-Cu(100)	
	E_a /eV	E_r /eV	E_a /eV	E_r /eV
$H_2O(g) + Cu_{Cu2O\text{-}Cu} \rightarrow H_2O_{Cu}$		-0.39		-0.70
$H_2O_{Cu} + O_{Cu2O} \rightarrow OH_{Cu} + O_{Cu2O}H$	0.58	0.36	0.40	0.33
$O_{Cu2O}H \rightarrow O_{Cu2O} + H_{Cu}$	0.98	0.27	1.09	0.43
	Cu(111)		Cu(100)	
	E_a/eV	E_r/eV	E_a/eV	E_r/eV
$H_2O + Cu_{Cu} \rightarrow H_2O_{Cu}$		-0.34		-0.37
$H_2O_{Cu} \rightarrow OH_{Cu} + H_{Cu}$	1.36	0.20	1.23	0.10
$OH_{Cu} \rightarrow O_{Cu} + H_{Cu}$	1.70	0.60	1.68	0.50

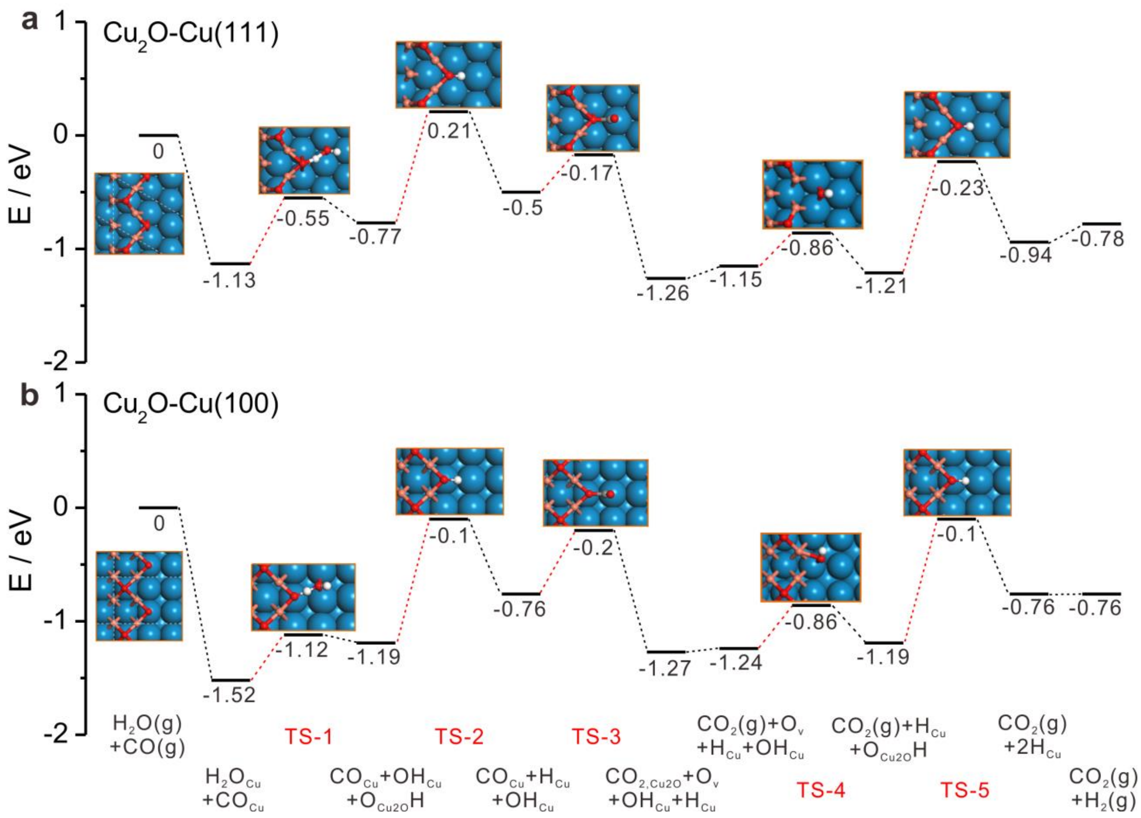

Supplementary Figure 20. Calculated energy diagram of the WGS reaction at the Cu_2O-Cu interfaces of (**a**) Cu(111) and (**b**) Cu(100) surfaces via the redox mechanism with optimized structures of involved key surface species and transition states.

Supplementary Table 3. The reaction energy (E_r) and reaction barrier (E_a) of elementary surface reactions of the WGS reaction catalyzed at the Cu_2O-Cu interfaces of Cu(111) and Cu(100) surfaces via the redox mechanism.

Elementary Reactions	Cu_2O-Cu(111)		Cu_2O-Cu(100)	
	E_a /eV	E_r /eV	E_a /eV	E_r /eV
$CO(g) + Cu_{Cu2O\text{-}Cu} \rightarrow CO_{Cu}$		-0.74		-0.82
$H_2O(g) + Cu_{Cu2O\text{-}Cu} \rightarrow H_2O_{Cu}$		-0.39		-0.70
$H_2O_{Cu} + O_{Cu2O} \rightarrow OH_{Cu} + O_{Cu2O}H$	0.58	0.36	0.40	0.33
$O_{Cu2O}H \rightarrow O_{Cu2O} + H_{Cu}$	0.98	0.27	1.09	0.43
$CO_{Cu} + O_{Cu2O} \rightarrow CO_{2,\ Cu2O} + O_v$	0.33	-0.76	0.56	-0.51
$OH_{Cu} + O_v \rightarrow O_{Cu2O}H$	0.29	-0.06	0.38	0.05
$O_{Cu2O}H \rightarrow O_{Cu2O} + H_{Cu}$	0.98	0.27	1.09	0.43
$H_{Cu} + H_{Cu} \rightarrow H_2(g)$	0.90	0.16	0.85	0
$CO_{2,\ Cu2O} \rightarrow CO_2(g)$		0.11		0.03

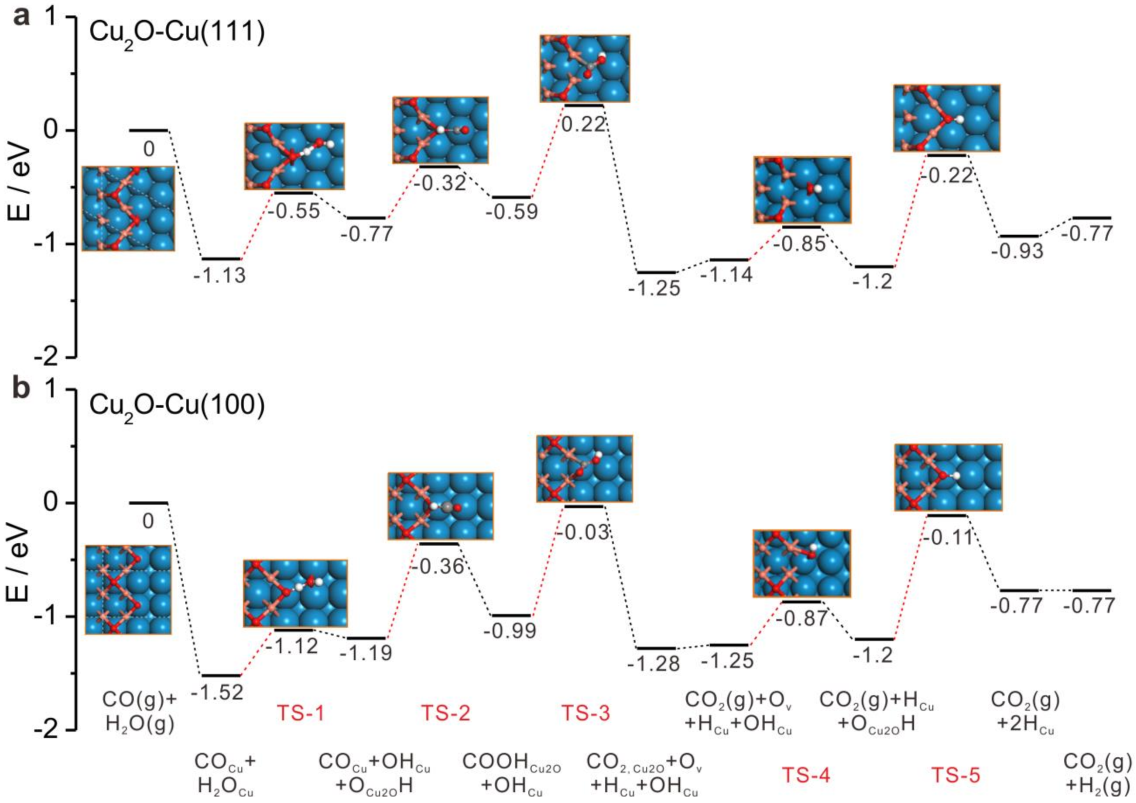

Supplementary Figure 21. Calculated energy diagram of the WGS reaction at the Cu_2O-Cu interfaces of (**a**) Cu(111) and (**b**) Cu(100) surfaces via the associative mechanism initiated by the reaction of CO_{Cu} and $O_{Cu2O}H$ to produce $COOH_{Cu2O}$ with optimized structures of involved key surface species and transition states.

Supplementary Table 4. The reaction energy (E_r) and reaction barrier (E_a) of elementary surface reactions of the WGS reaction catalyzed at the Cu_2O-Cu interfaces of Cu(111) and Cu(100) surfaces via the associative mechanism initiated by the reaction of CO_{Cu} and $O_{Cu2O}H$ to produce $COOH_{Cu2O}$.

Elementary Reactions	Cu_2O-Cu(111)		Cu_2O-Cu(100)	
	E_a /eV	E_r /eV	E_a /eV	E_r /eV
$CO(g) + Cu_{Cu2O\text{-}Cu} \rightarrow CO_{Cu}$		-0.74		-0.82
$H_2O(g) + Cu_{Cu2O\text{-}Cu} \rightarrow H_2O_{Cu}$		-0.39		-0.70
$H_2O_{Cu} + O_{Cu2O} \rightarrow OH_{Cu} + O_{Cu2O}H$	0.58	0.36	0.40	0.33
$CO_{Cu} + O_{Cu2O}H \rightarrow COOH_{Cu2O}$	0.45	0.18	0.83	0.20
$COOH_{Cu2O} \rightarrow CO_{2,\ Cu2O} + H_{Cu}+O_v$	0.81	-0.66	0.96	-0.29
$OH_{Cu} + O_v \rightarrow O_{Cu2O}H$	0.29	-0.06	0.38	0.05
$O_{Cu2O}H \rightarrow O_{Cu2O} + H_{Cu}$	0.98	0.27	1.09	0.43
$H_{Cu} + H_{Cu} \rightarrow H_2(g)$	0.90	0.16	0.85	0
$CO_{2,\ Cu2O} \rightarrow CO_2(g)$		0.11		0.03
$CO_{2,\ Cu2O} + H_{Cu} \rightarrow HCOO_{Cu2O}$	0.81	-0.66	0.49	-0.46
$HCOO_{Cu2O} \rightarrow CO_{2,\ Cu2O} + H_{Cu}$	1.47	0.66	0.95	0.46

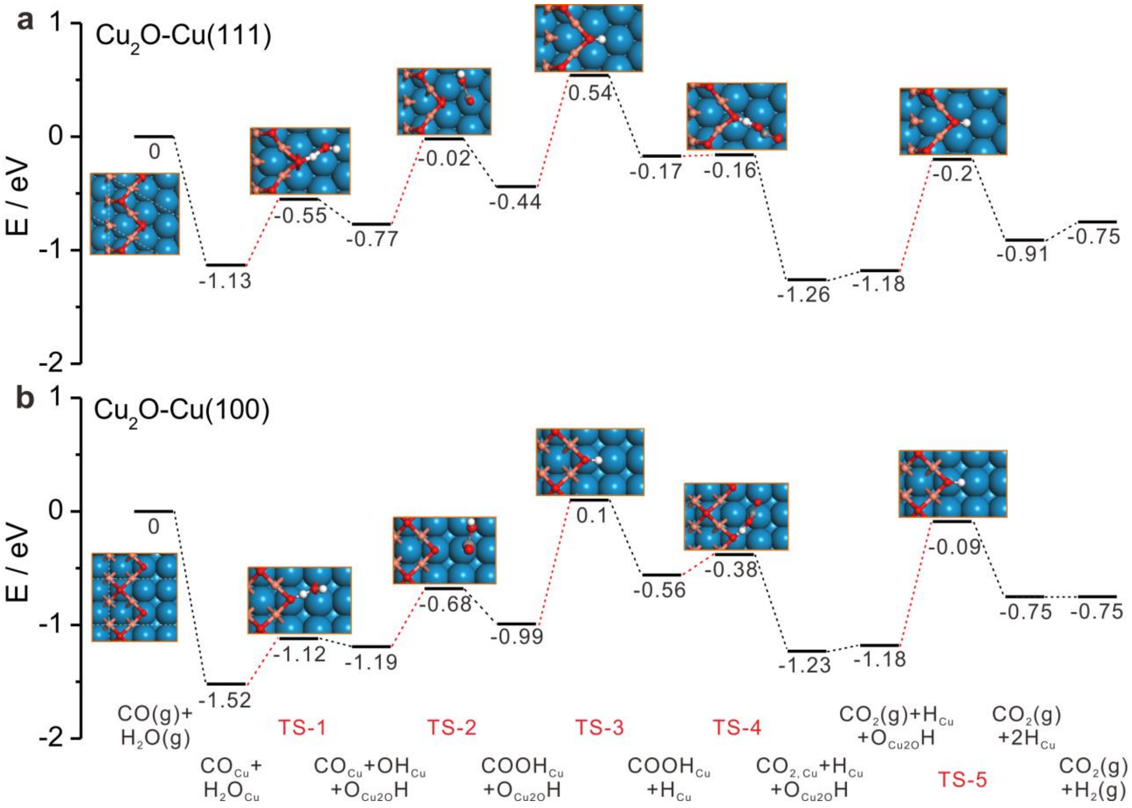

Supplementary Figure 22. Calculated energy diagram of the WGS reaction at the Cu_2O-Cu interfaces of (**a**) Cu(111) and (**b**) Cu(100) surfaces via the associative mechanism initiated by the reaction of CO_{Cu} and OH_{Cu} to produce $COOH_{Cu}$ with optimized structures of involved key surface species and transition states.

Supplementary Table 5. The reaction energy (E_r) and reaction barrier (E_a) of elementary surface reactions of the WGS reaction catalyzed at the Cu_2O-Cu interfaces of Cu(111) and Cu(100) surfaces via the associative mechanism initiated by the reaction of CO_{Cu} and OH_{Cu} to produce $COOH_{Cu}$.

Elementary Reactions	Cu_2O-Cu(111)		Cu_2O-Cu(100)	
	E_a /eV	E_r /eV	E_a /eV	E_r /eV
$CO(g) + Cu_{Cu2O-Cu} \rightarrow CO_{Cu}$		-0.74		-0.82
$H_2O(g) + Cu_{Cu2O-Cu} \rightarrow H_2O_{Cu}$		-0.39		-0.70
$H_2O_{Cu} + O_{Cu2O} \rightarrow OH_{Cu} + O_{Cu2O}H$	0.58	0.36	0.40	0.33
$CO_{Cu} + OH_{Cu} \rightarrow COOH_{Cu}$	0.75	0.33	0.51	0.20
$O_{Cu2O}H \rightarrow O_{Cu2O} + H_{Cu}$	0.98	0.27	1.09	0.43
$COOH_{Cu} + O_{Cu2O} \rightarrow CO_{2, Cu} + O_{Cu2O}H$	0.01	-1.09	0.18	-0.67
$O_{Cu2O}H \rightarrow O_{Cu2O} + H_{Cu}$	0.98	0.27	1.09	0.43
$H_{Cu} + H_{Cu} \rightarrow H_2(g)$	0.90	0.16	0.85	0
$CO_{2, Cu} \rightarrow CO_2(g)$		0.08		0.05
$CO_{2, Cu} + H_{Cu} \rightarrow HCOO_{Cu}$	0.75	-0.51	0.53	-0.74
$HCOO_{Cu} \rightarrow CO_{2, Cu} + H_{Cu}$	1.26	0.51	1.27	0.74

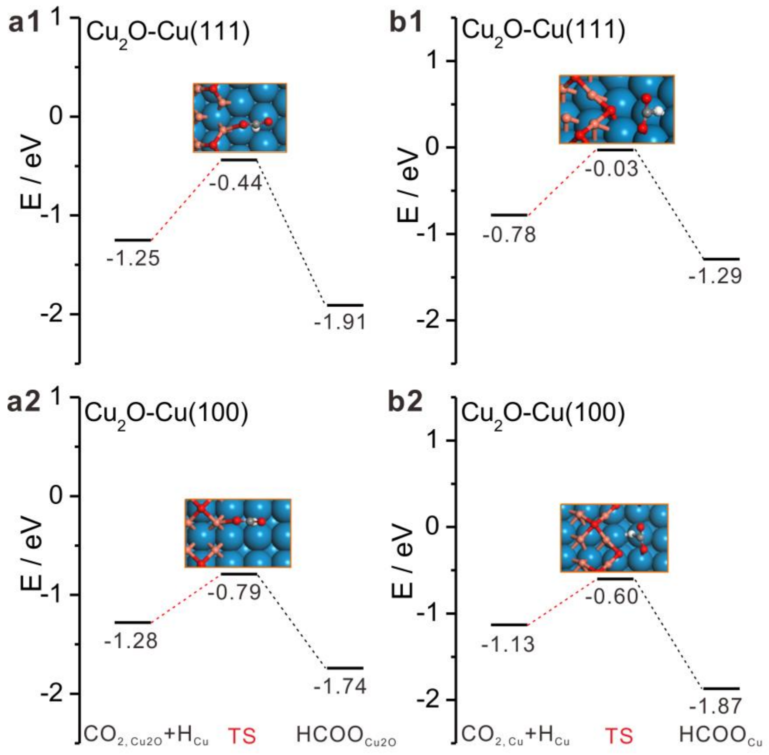

Supplementary Figure 23.Calculated energy diagram of the reaction of adsorbed CO_2 and H to form the formate species at the Cu_2O-Cu interfaces of (**a1, b1**) Cu(111) and (**a2, b2**) Cu(100) surfaces with optimized structures of the transition state.

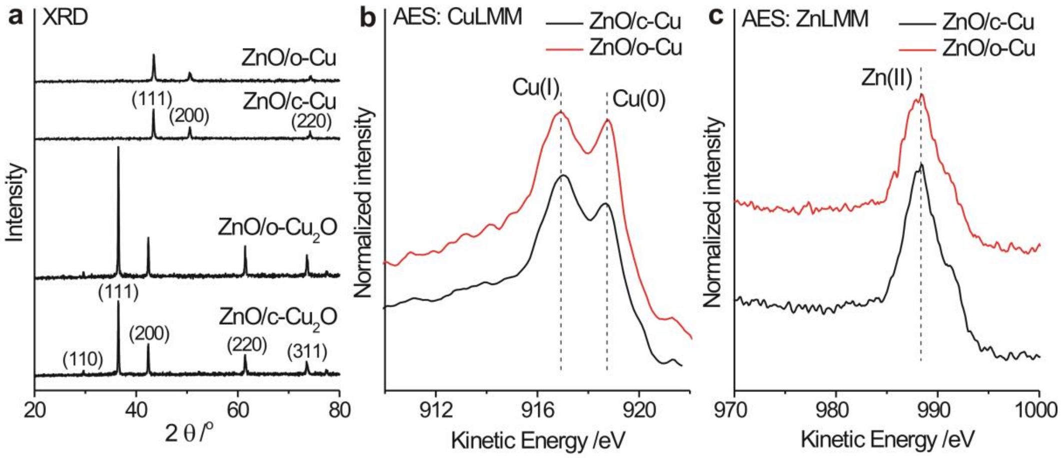

Supplementary Figure 24. (**a**) XRD patterns, (**b**) Cu LMM and (**c**) Zn LMM AES spectra of ZnO/Cu_2O-NC and ZnO/Cu-NC catalysts.

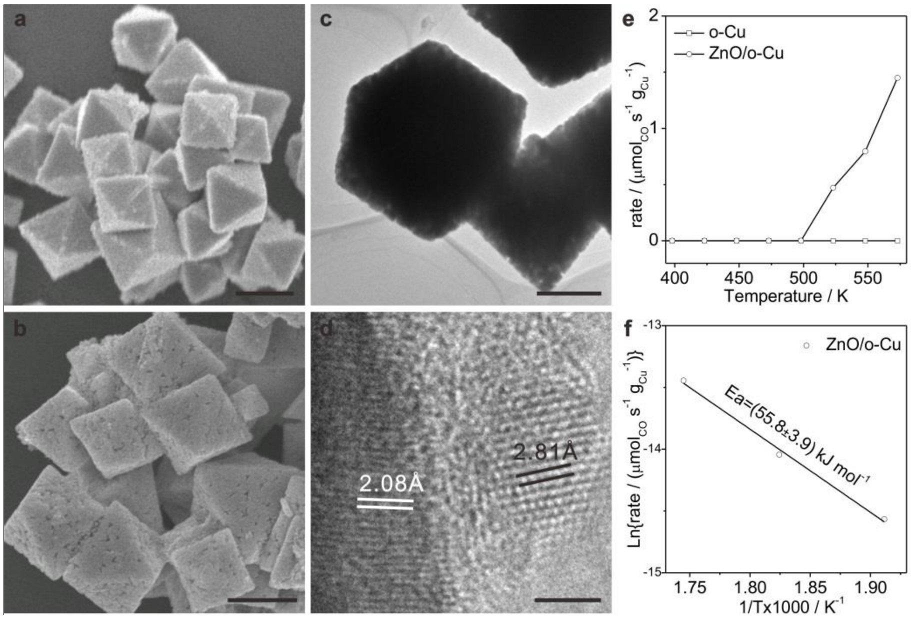

Supplementary Figure 25. ZnO/o-Cu catalyst. The scale bars of (**a, b**) correspond to 1000 nm, that of (**c**) correspond to 500 nm, and that of (**d**) correspond to 2 nm. Representative SEM images of (**a**) 1%wt-ZnO/o-Cu_2O, representative SEM (**b**), TEM (**c**) and HRTEM (**d**) images of 1.67%wt-ZnO/o-Cu catalysts, (**e**) CO conversions of 1.67%wt-ZnO/o-Cu catalyst in the WGS reaction as a function of reaction temperature and (**f**) Arrhenius plot of 1.67%wt-ZnO/o-Cu in the WGS reaction. The data of o-Cu NCs are included for comparisons. The lattice fringes of 2.08 and 2.81 Ǻ respectively correspond to the spacing of Cu {200} (JCPDS card NO. 89-2838) and hexagonal ZnO{100}(JCPDS card NO 89-1397) crystal planes.

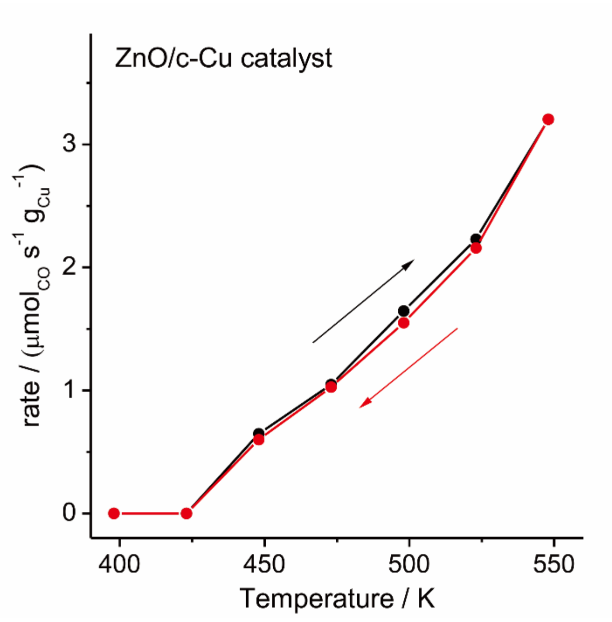

Supplementary Figure 26. Catalytic performance of ZnO/c-Cu catalyst in the WGS reaction evaluated with cycling reaction temperatures. The observed reproducibility demonstrated the stability of ZnO/c-Cu catalyst in the WGS reaction up to 548 K.

Supplementary References:

[1] Horn, K., Pritchard, J. Infrared spectrum of CO chemisorbed on Cu (100). *Surf. Sci.* **55**, 701-704 (1976).

[2] Fogler, H. S. Elements of chemical reaction engineering. Prentice Hall Professional Technical Reference, 2006.

[3] Vannice, M. A. Kinetics of catalytic reactions. Springer Science + Business Media, Inc. 2005.

[4] Perry, R., Green, D. Perry's chemical engineers' handbook. McGraw-Hill Education, 2008.

[5] Kresse, G., Joubert, D. From ultrasoft pseudopotentials to the projector augmented-wave method. *Phys. Rev. B* **59**, 1758 (1999).

[6] Kresse, G., Furthmuller, J. Efficient iterative schemes for ab initio total-energy calculations using a plane-wave basis set. *Phys. Rev. B* **54**, 11169 (1996).

[7] Blochl, P. E. Projector augmented-wave method. *Phys. Rev. B* **50**, 17953 (1994).

[8] Perdew, J. P., Wang, Y. Pair-distribution function and its coupling-constant average for the spin-polarized electron gas. *Phys. Rev. B* **45**, 13244 (1992).

[9] Perdew, J. P., Burke, K., Ernzerhof, M. Generalized gradient approximation made simple. *Phys. Rev. Lett.* **77**, 3865 (1996).

[10] Mishin, Y., Mehl, M. J., Papaconstantopoulos, D. A., Voter, A. F., Kress, J. D. Structural stability and lattice defects in copper: Ab initio, tight-binding, and embedded-atom calculations. *Phys. Rev. B* **63**, 224106 (2001).

[11] Besenbacher, F., Nørskov, J.K. Oxygen chemisorption on metal surfaces: general trends for Cu, Ni and Ag. *Prog. Surf. Sci.* **44**, 5-66 (1993).

[12] Matsumoto, T., Bennett, R. A., Stone, P., Yamada, T., Domen, K., Bowker, M. Scanning tunneling microscopy studies of oxygen adsorption on Cu(111). *Surf. Sci.* **471**, 225-245 (2001).

[13] Soon, A., Todorova, M., Delley, B., Stampfl, C. Oxygen adsorption and stability of surface oxides on Cu(111): a first-principles investigation. *Phys. Rev. B* **73**, 165424-165434 (2006).

[14] Yang, F., Choi, Y., Liu, P., Hrbek, J., Rodriguez, J. A. Autocatalytic reduction of a Cu_2O/Cu(111) surface by CO: STM, XPS, and DFT studies. *J. Phys. Chem. C* **114**, 17042-17050 (2010).

[15] Henkelman, G., Jonsson, H. Improved tangent estimate in the nudged elastic band method for finding minimum energy paths and saddle points. *J. Chem. Phys.* **113**, 9978 (2000).

[16] Henkelman, G., Uberuaga, B. P., Jonsson, H. A climbing image nudged elastic band method for finding saddle points and minimum energy paths. *J. Chem. Phys.* **113**, 9901 (2000).

[17] Sun, K., Zhao, Y., Su, H., Li, W. Force reversed method for locating transition states. *Theor. Chem. Acc.* **131**, 1118 (2012).
